# The History and Innovations of Blood Vessel Anastomosis

**DOI:** 10.3390/bioengineering9020075

**Published:** 2022-02-15

**Authors:** William R. Moritz, Shreya Raman, Sydney Pessin, Cameron Martin, Xiaowei Li, Amanda Westman, Justin M. Sacks

**Affiliations:** 1 Division of Plastic and Reconstructive Surgery, Department of Surgery, Washington University School of Medicine, St. Louis, MO 63110, USA; moritzwr@wustl.edu (W.R.M.); 23sspessin@ecfs.org (S.P.); cameronm@wustl.edu (C.M.); xiaoweili@wustl.edu (X.L.); a.m.westman@wustl.edu (A.W.); 2 School of Medicine, Virginia Commonwealth University, Richmond, VA 23298, USA; sraman3@vcu.edu

**Keywords:** surgical history, anastomosis, innovation, surgical technology, microsurgery, vascular surgery

## Abstract

Surgical technique and technology frequently coevolve. The brief history of blood vessel anastomosis is full of famous names. While the techniques pioneered by these surgeons have been well described, the technology that facilitated their advancements and their inventors deserve recognition. The mass production of laboratory microscopes in the mid-1800s allowed for an explosion of interest in tissue histology. This improved understanding of vascular physiology and thrombosis laid the groundwork for Carrel and Guthrie to report some of the first successful vascular anastomoses. In 1916, McLean discovered heparin. Twenty-four years later, Gordon Murray found that it could prevent thrombosis when performing end-to-end anastomosis. These discoveries paved the way for the first-in-human kidney transplantations. Otolaryngologists Nylen and Holmgren were the first to bring the laboratory microscope into the operating room, but Jacobson was the first to apply these techniques to microvascular anastomosis. His first successful attempt in 1960 and the subsequent development of microsurgical tools allowed for an explosion of interest in microsurgery, and several decades of innovation followed. Today, new advancements promise to make microvascular and vascular surgery faster, cheaper, and safer for patients. The future of surgery will always be inextricably tied to the creativity and vision of its innovators.

## 1. Introduction

Achieving successful anastomosis of blood vessels is critical for many modern surgical procedures. Today, organ transplantations, vascular repair after trauma, and free tissue transfer under the microscope are all commonplace. However, only 100 years ago, vascular anastomosis was something that could only be achieved in the laboratory setting. By the time Alexis Carrel was awarded the Nobel Prize in 1912 for his work connecting vessels together, a new door had been opened to the future of surgery. While we frequently know the names of the surgeons who took us to our present era of microvascular surgery—Carrel, Murray, Jacobson, Buncke, Tamai, Koshima—it is important to recognize the technological advances that made their success possible. In this paper, we hope to explore the history of vascular anastomosis through the lens of technological innovation. We will describe the origins of microscopy, the discovery of heparin, and the development of the first microsurgical tools. We hope that through this article, the reader will appreciate that no surgical innovation can be separated from technological advancement.

## 2. Early Vascular Repair (before 1900)

Prior to the early 1900s, ligation was the sole method of repairing vascular injuries. Ligation was described intermittently in the pre-modern world. Writing sometime between the fourth and seventh centuries AD, Byzantine surgeon Paul of Aegina described ligation for the management of aneurysms, varicose veins, and wartime arterial injuries [1,2]. His works would continue to influence Arabian medical texts throughout the Middle Ages. Henri de Mondeville, born 1260 in Normandy, France further described the use of ligation for managing vascular injuries [3]. However, ligation was not widely accepted by the European medical community for another 300 years, when Ambroise Paré, a French military surgeon, reintroduced the idea for managing wartime vascular injuries. Before Paré, amputations were common but had a high mortality rate due to severe blood loss. As a solution, Paré reintroduced the idea of vessel ligation using a small thread-like or wire material [4]. While vessel ligation was a critical advancement for surgeons at the time, there remained a belief that injured vessels could be repaired primarily, restoring blood flow to distal structures. 

In a 1761 letter to Dr. William Hunter, Richard Lambert of Newcastle upon Tyne reported a successful repair of a small tear in the brachial artery by approximating the wound edges with a small steel pin. However, his results could not be replicated, and attempts at vascular anastomosis were shelved for over a century [5]. Understandings of vessel physiology and thrombosis were primitive at the time and likely contributed to these failed efforts. Advances in microscopy and tissue histology were needed before vascular anastomosis could be attempted.

## 3. The Birth of Microscopy and Early Techniques (1850s to 1940s)

Optics and magnification underwent a period of growth during the 19th century, when both surgical loupes and laboratory microscopes were developed for widespread use.

In 1846, German machinist Carl Zeiss opened a workshop in Jena, Germany to repair optical and scientific instruments [6]. He created his first microscope in 1847, and by 1866, he had produced more than 1000 of them. However, recognizing the need to bring experienced scientists to his lab to improve his microscopes, he hired a professor of physics from Jena University, Ernst Abbe, as Director of Research in 1866. By employing his own equation, which would become known as ‘Abbe’s Sine Condition’, Abbe was able to consistently produce accurate, standardized lenses for every microscope created by the company. This allowed for mass production and, eventually, mass utilization of Zeiss microscopes [6]. 

Almost simultaneously, loupes were invented by French optician Charles Louis Chevalier in the 1840s [6]. Edwin Theodore Saemich of Bonn then adapted the design for surgery in 1876 [6,7]. In the early 20th century, Moritz van Rohr engineered a lighter set of loupes that were ultimately manufactured and distributed with the help of Carl Zeiss. The use of optical loupes allowed human tissue to be visualized with greater acuity. 

Increased access to laboratory microscopes, as well as the development of new staining and tissue-fixation techniques, led to an explosion of interest in tissue histology in the mid-to-late 1800s [8]. During this period, microscopy was integrated with medicine, as scientists used these techniques to develop a greater understanding of disease pathogenesis [8]. When applied to blood vessels, microscopy led to a greater understanding of blood vessel microanatomy and physiology. Histological studies of thrombosed vessels—as well as clinical observations made by surgeons like Virchow, Paget, and Billroth—drastically improved the understanding of the thrombotic cascade. By the end of the 19th century, it was understood that preservation of the endothelium was important for preventing thrombosis and that infection of the surgical site could result in coagulation [9].

Surgical loupes would eventually go on to become a critical tool in vascular surgery, but first laboratory science would need to be integrated into surgical technique. With improved tools and a growing body of knowledge, surgeons were poised to complete the first successful vascular anastomosis.

### 3.1. First Successful Blood Vessel Anastomoses

**Figure 1:**
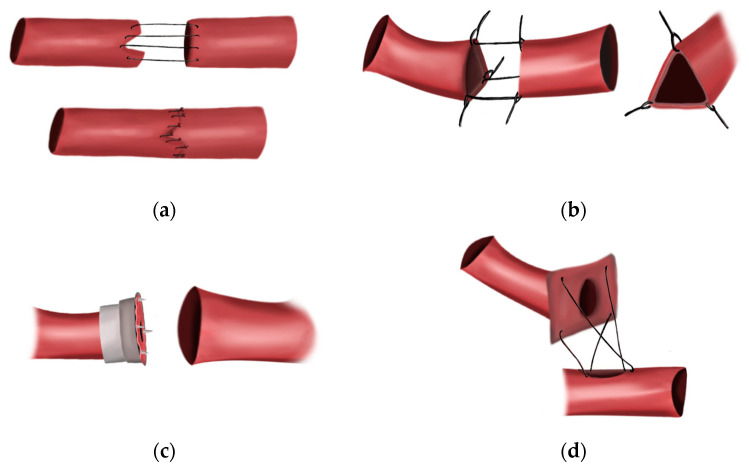
The early methods of vascular anastomosis. (**a**) The invagination technique developed by J.B. Murphy in 1897. (**b**) The triangulation technique developed by Carrel in 1902. Special care was taken to avoid piercing the intima. (**c**) Payr’s sutureless anastomotic device designed in 1904. (**d**) The patch technique developed by Guthrie and Carrel in 1906. Surgeons no longer avoided piercing the intima with their stitches.

In 1889, Alexander Jassinowsky theorized that it would one day be possible to repair vascular injuries. Two years later, he reported 26 successful arterial repairs in vessels of varying sizes in animals [5]. In 1897, J.B. Murphy in the United States utilized the current medical understanding of aseptic technique and vessel physiology to build on the preliminary success of Jassinowsky and develop a new technical framework for blood vessel anastomosis [5,9]. He utilized the histomechanical principle of Thoma—which states that as vessel caliber is decreased, vessel wall tension decreases—to develop the invagination method of vascular anastomosis, which found some success in his animal models (Figure 1a) [9]. He also emphasized the importance of infection and desiccation prevention, as well as accurate vessel-wall approximation to prevent thrombosis. However, Murphy, like Jassinowsky, believed that damaging the tunica intima would lead to coagulation, a belief that would be shown to be the final barrier to consistent success with their techniques [9].

The publication of the triangulation technique for end-to-end anastomosis by Alexis Carrel—considered by many to be the father of vascular surgery—in 1902 was the next significant breakthrough [10]. In the triangulation method, three stitches, each placed one-third of the way around the cut end of a vessel, could be used to retract a vessel and form a triangle (Figure 1b). By approximating two triangulated vessels, continuous suturing could be performed, allowing for the first true end-to-end anastomosis [10]. However, this technique produced limited success, and it was not until Carrel moved to America and began working in the laboratory of Charles Claud Guthrie that his technique was perfected. 

Guthrie applied many of the same techniques espoused by J.B. Murphy, insisting on rigorous aseptic technique and judicious application of vaseline to the vessels to prevent thrombosis [11]. However, unlike Carrel, Murphy, and Jassinowsky, Guthrie insisted that vessel approximation could only be achieved by including, rather than excluding, the tunica intima. While Guthrie did not know it at the time, this small change in technique was critical in preventing anastomotic thrombosis. By using this technique, Guthrie approximated intima to intima, avoiding exposure of subendothelial collagen and tissue factor to circulating factor VII, preventing activation of the extrinsic coagulation cascade. This discovery allowed for more consistent anastomotic success. In 1906, Carrel and Guthrie published the “patch method” to accomplish closure of openings in vessel walls by anastomosing a vessel to another vessel, or even other structures [12]. The “patch method” involved dissecting the vessel of origin in a manner that allowed the edges to be splayed open, with the vessel lumen in the center (Figure 1c). The “patch” could then be sutured to a created opening in the wall of another vessel, essentially creating a terminolateral anastomosis [12]. In the initial experiments, Carrel and Guthrie were able to dissect and remove the spermatic artery of a dog and anastomose it to the femoral artery [12]. Through their larger experiment, they performed this technique in 14 cases of transplantation of the kidney and the ovaries in cats and dogs by patching the vessels to veins or peritonea [12]. Infection occurred often, though they noted that blood flow was satisfactory through the organs of interest. Through this initial experiment, Carrel and Guthrie concluded that the patch method was successful in anastomoses, and even remained patent four months following the operation [12]. Carrel was eventually awarded the Nobel Prize in 1912, whereas Guthrie’s contributions were largely forgotten by the medical community [10,11,13].

### 3.2. First Interposition Vein Grafts

Shortly after the first experimental vascular anastomoses were performed, attempts were made to bring these techniques into the clinical setting. The first recorded interposition vein graft was performed in 1906 by Jose Goyanes using everting sutures with through-and-through stitches. The patient recovered well after a minor infection at the surgical site [14,15]. J. Hogarth Pringle then published two successful attempts at the same procedure using the techniques developed by Carrel and Guthrie [16].

### 3.3. Sutureless Anastomosis

Advancements in sutureless anastomosis paralleled these early innovations in sutured anastomosis. In 1900, Erwin Payr designed a magnesium ring that could assist in anastomosis by threading the proximal vessel through the ring, everting the edges over the circular ring, and inserting it within a dilated distal end of the vessel [17]. In 1904, Payr innovated on his own idea by adding pins orthogonal to the circular interface of the rings in order to achieve more stability when hooking the edges of the vessels during eversion [17].

Although these early attempts at vascular anastomosis were successful in the laboratory, results in the operating room were limited. In World War I, 4404 US soldiers lost one or more extremity. Of these, only 13% lost their limbs on the battlefield. The remaining 3713 amputations occurred in the operating room due to infection or serious damage to blood supply. Attempts at reconnecting damaged arteries were endeavored but were frequently unsuccessful [18]. Further innovation was required to ensure consistent success.

### 3.4. Discovery of Heparin

**Figure 2:**
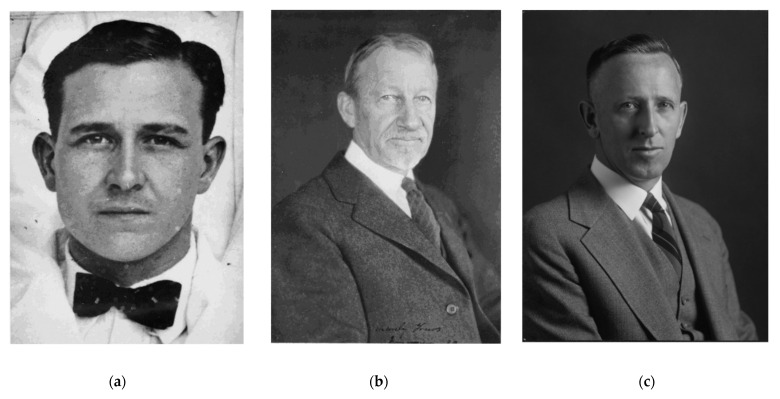
Important figures in the history of heparin. (**a**) Jay McLean. As a medical student, McLean isolated heparin from liver extracts [25]. (**b**) William Henry Howell. The professor at Johns Hopkins University who hired McLean and worked to characterize the compound until his death in the 1940s [25]. (**c**) Gordon Murray. In 1940, Murray described the first use of heparin as an adjunct to end-to-end anastomosis in the clinical setting. His repair of a lacerated brachial artery was successful [24].

Incredibly, the successful vascular anastomoses reported by these early surgeons were achieved without the use of microscopes or anticoagulants. It was not until 1916 that Johns Hopkins medical student Jay McLean (Figure 2a) isolated an antithrombogenic phosphatide while working in the lab of Professor William Howell [19,20]. This compound, which was eventually named heparin, was found to completely prevent coagulation in solutions as dilute as 0.1% [21]. Although Howell originally thought heparin acted to increase prothrombin time in the years following its first characterization, later studies found that it actually increased partial thromboplastin time. In 1929, heparin was found to reduce the incidence of thrombosis after mechanical or chemical injury to the intimal surfaces of peripheral veins [22]. In 1937, heparin was made available for commercial use. In 1940, Gordon Murray (Figure 2c) further showed that heparin could greatly reduce the incidence of thrombosis in both arterial anastomoses and vein grafts, improving incidence of patency from 35–80% in an experimental model of brachial and femoral anastomosis [23,24]. In this same paper, Murray described the use of heparin in the repair of a traumatic injury to the brachial artery. He reported that the injury, which would have been managed with ligation at the time, was successfully repaired with an end-to-end anastomosis [23]. While the first successes of surgeons, such as Murphy, Carrel, and Guthrie, were a testament to their incredible care and attention to technique, it was the isolation of heparin that allowed for successful vascular anastomosis to be achieved broadly by all surgeons and with more consistent results.

### 3.5. First Clinical Applications of Vascular Anastomosis

In 1943, Arthur Blakemore described a new sutureless technique for achieving vascular repair that would eventually be implemented in the Second World War on a small scale. He lined a Vitallium (65% cobalt, 30% chromium, 5% molybdenum) tube with a vein graft and used the tube to connect the two cut ends of the damaged vessel. At the time, vein grafts were the only practical method of anastomosing arteries, and poor outcomes after attempts at sutured anastomosis in the previous world war indicated a need for a method that avoided sutures altogether in the combat theater [18,26].

In 1950, using an end-to-end suturing technique and heparin injected into the renal artery and vein, Richard Lawler performed the first kidney transplantation in a human. The anastomosis remained patent for 63 days, when a nephrectomy was performed due to ureteral stricture [27]. Six years later, Joseph Murray reported the first successful renal transplantation, connecting the renal vessels of the donor kidney to the recipient iliac vessels in an end-to-side fashion. He was eventually awarded the Nobel Prize for his work [28].

This achievement was the culmination of over 50 years of technological advances, with each new discovery pushing surgeons closer to achieving consistent success with vascular anastomosis in the clinical setting. While these techniques allowed for suturing of large vessels, vessels smaller than 4 mm could not be connected. It was not until a second technological revolution that this size barrier could be breached.

## 4. The Birth of Microsurgery

**Figure 3:**
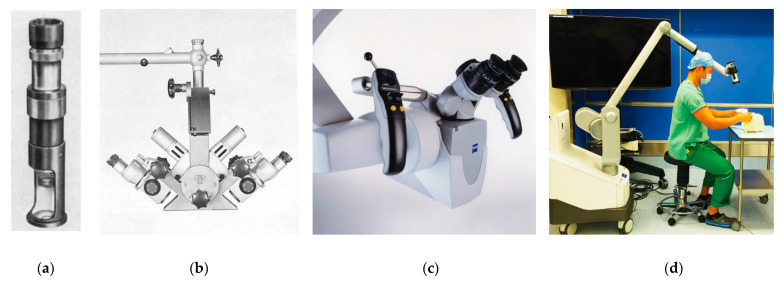
The evolution of the operative microscope. (**a**) The Brinell-Leitz microscope, used by Carl Nylen in 1921 to perform the first microsurgery [29]. (**b**) The Zeiss diploscope, developed by Hans Littman at the request of Julius Jacobson, used beam-splitting technology developed during the Second World War. It was the first microscope that allowed the surgeon and one assistant to view the operative field simultaneously. Source: Zeiss Archives. (**c**) The Zeiss OPMI Pentero, a modern operative microscope that allows for fluorescent imaging in situ. Source: Zeiss Archives. (**d**) The Olympus ORBEYE three-dimensional exoscope, a new alternative that provides a real-time, high-definition, and magnified video feed to the entire surgical team [31].

While microscopes were increasingly utilized in the laboratory in the late 19th and early 20th century, it was not until 1921 that the microscope was brought into the operating room [7]. That year, Carl Nylen, an otolaryngologist in Stockholm, Sweden decided to use a monocular Brinell-Leitz microscope to treat a patient with chronic otitis media (Figure 3a) [29]. The following year, Gunnar Holmgren of the same institution adapted a binocular microscope with an attached light source for the same procedure, overcoming issues with depth perception and poor lighting encountered by Nylen [30]. A period of optimization followed over the next three decades, culminating in the creation of the Zeiss OPMI-1 by Hans Littman in 1953 [6]. This microscope addressed two critical problems that had slowed microsurgery’s broad adoption: poor lighting and a lack of stable, flexible support systems [7]. The OPMI-1 was mounted on a rotating arm and was equipped with coaxial lighting sources. Furthermore, it allowed surgeons to change magnification without altering focal length, a critical development that greatly facilitated its use in the operating room [6].

### 4.1. First Microvascular Surgery

By the time Julius Jacobson joined the faculty at the University of Vermont as an Associate Professor and Director of Surgical Research, otologists and ophthalmologists had already been incorporating microscopes into surgical procedures for decades [7]. However, in 1960, microscopes had not yet been used in vascular surgery. Early in his tenure, Jacobson was asked to collaborate on a project with the Department of Pharmacology to study the effects of drugs in canines with denervated carotid arteries [32,33,34,35]. He postulated that the only way to achieve complete denervation was by transecting the artery and rejoining the cut ends. To do this, Jacobson had to identify and address the issues that had kept surgeons from consistently connecting vessels smaller than 4 mm. Believing that magnification of the operative field could help him approximate the vessels and minimize trauma, Jacobson decided to borrow an operative microscope from the otolaryngologists at Mary Fletcher Hospital in Burlington, Vermont [34,35]. That same day, he successfully performed the first canine carotid anastomosis under a microscope [36]. The consistency of his results using the microscope were striking: arteries connected under the microscope had a 100% patency rate, whereas those connected without magnification remained patent only 70% of the time [37]. Working with Vermont neurosurgeon R.M.P Donaghy, Jacobson applied his microsurgical techniques to humans for the first time. In 1962, Donaghy and Jacobson reported the world’s first microneurovascular surgery by performing a middle cerebral artery embolectomy [38,39].

### 4.2. Development of Microsurgical Tools

The creation of this new specialty required further innovation and new instruments. Suture materials and microscopes had to be tailored to facilitate the level of precision and visualization demanded by these procedures. 

#### 4.2.1. Needle Holders

In 1951, Ramon Castroviejo developed the first spring-handle needle holders for microsurgery by designing the device to facilitate the fine finger movements—rather than wrist actions—required for ophthalmological microsurgery [40]. Julius Jacobson adapted these devices to his deeper microvascular surgeries and obtained jeweler forceps and scissors from a store in Burlington, Vermont, USA [34,35].

#### 4.2.2. Suture Material

With these finer instruments, smaller needles and suture material also needed to be developed. At the time, the finest suture material available was 6-0. Ethicon created a needle 5/1000 ths of an inch for Jacobson and found a machinist capable of drilling a 1/1000 th inch hole in its stock, allowing for the finest suture material available to be swaged [34,35].

#### 4.2.3. Vascular Clamps

While vascular clamps had been developed for larger vessel procedures, they needed to be miniaturized for microsurgery. In 1948, Willis J. Potts, a pediatric surgeon, devised vascular clamps for the treatment of patent ductus arteriosus [41]. These clamps were equipped with fine teeth and a locking mechanism that prevented the clamp from being closed completely, protecting the vessel. Jacobson adopted these clamps in his own microsurgical work [34]. In the 1950s, Frank Mayfield developed his eponymous cross-legged clips [42]. Although Mayfield originally intended them for the management of intracranial aneurysms, they were also applied for temporary arterial occlusion and found utility in microvascular surgery [43,44]. Susumu Tamai handmade his own microsurgical clamps with two Scoville–Lewis clips and a 22-gauge needle [45]. However, the Mayfield and Scoville–Lewis clips were bulky and hard to apply, tending to shift in the operative field from their own weight [44]. To resolve this issue, Robert Acland developed his own clamps for microvascular surgery in 1972. Lightweight and easily applied with surgical forceps, these clamps were well suited for their role and are still in use today [44].

#### 4.2.4. Electrocautery

In order to provide hemostasis and keep the surgical field free of blood, a new electrocautery device needed to be developed that could minimize trauma in the miniaturized surgical field. The original monopolar cautery was developed by Harvey Cushing and a physicist named William T. Bovie in 1926 [46]. However, its use could result in damage to the local anatomy due to uncontrolled discharge of the current. In the 1940s, James Greenwood from the Methodist Hospital in Houston, Texas engineered the bipolar coagulator to address this problem [46]. The first commercial bipolar coagulator was made in 1955 through the design of Dr. Leonard Malis [46]. By adding a damped-wave spark unit, he was able to achieve nearly zero current leakage from the forceps tips into the surrounding anatomy [47]. Settings on the unit were also introduced to adjust the strength of the bipolar from a level of 1 to 10, where level 1 could be used for very fine microvascular work [47]. He was also the first to note its potential application for use with skin flaps in plastic surgery, though his use of the bipolar was primarily in his neurosurgical procedures [47].

#### 4.2.5. Venous Coupler

Incidentally, sutureless anastomosis was introduced to microvascular surgery very early in its history. In 1962, Nakayama obtained the patent for metallic rings that used metal pins to hold the cut ends of vessels together with eversion [17,48]. In his patent, Nakayama demonstrated the device’s ability to approximate vessels between 1.5–4 mm in diameter. However, like much of the history of vascular anastomosis, credit was not fairly distributed. In his patent, Nakayama cited an image of a similar-looking device invented by a then-student at Northwestern University, Gordon P. Holt [48]. Prior to 1961, Holt performed 18 successful femoral artery anastomoses in dogs with his device. In 1961, the Chicago American newspaper reported on Holt’s work. However, since his name was not mentioned in Nakayama’s patent, Holt’s contributions went largely unrecognized by history [48].

#### 4.2.6. Double-Binocular Microscope

Finally, a microscope needed to be developed that would account for the specific demands of microvascular surgery. This final major advancement came from Hans Littman, the original creator for the Zeiss OPMI-1 microscope. In 1964, he agreed to make Jacobson a single double-binocular microscope [6]. This microscope, eventually named the Zeiss Diploscope, employed beam-splitting technology developed during World War II so a second surgeon could assist the primary surgeon by viewing the same operating field [38]. The first of these microscopes went to Jacobson; the second to Jim Smith, who was the first to apply the microscope to peripheral nerve repair [49]; and the third went to Harold Buncke in San Mateo, CA, USA [7]. These innovations paved the way for an explosion of interest in microvascular surgery (Table 1).

### 4.3. Development of Supermicrosurgery

Due to the knowledge of cutaneous vascular anatomy at the time, microvascular surgeons performing free tissue transfer were limited to musculocutaneous flaps until the 1980s. In 1987, Taylor and Palmer mapped the angiosomes of the body in incredible detail [62]. By identifying the vascular territories of these perforating vessels, surgeons were able to design new flaps that minimized donor site morbidity. In 1989, Koshima reported the first free flap based on perforating vessels, the deep inferior epigastric perforator flap [32,63]. However, this procedure required careful dissection of the perforating vessels through the rectus abdominis. Desiring to reduce time spent dissecting perforating vessels, Koshima designed flaps based on smaller and more distal vessels [64,65]. These vessels were between 0.8 and 0.3 mm in diameter, a size that had been attempted once before 1980—by Pennington and Pelly, without success. To accomplish the level of precision required for vessels of these size, delicate 30 µm needles were developed [66,67]. By reporting vascularized toenail transfers for fingernail losses [68] and distal-most fingertip reconstructions [69], Koshima demonstrated the power of his technique.

Today, supermicrosurgery is on the leading edge of reconstructive surgery. The techniques developed by Koshima and his colleagues have opened the door for surgical treatments that would have been science fiction to Murphy, Carrel, and Guthrie. The technique has allowed for lymphovenous bypass and free lymph node transfer for lymphedema [70], vascularized nerve grafts for segmental peripheral nerve injuries [66], and the development of new flaps for free tissue transfer. The benefits of supermicrosurgery are numerous, allowing for reduced operative time and minimized donor site morbidity [70]. However, supermicrosurgery requires significant skill, as well as specialized instruments and microscopes [70]. Innovation will be required for supermicrosurgery to become accessible to all patients and providers

## 5. New Directions and Outlook

### 5.1. Exoscopes

During the present era, operating microscopes have become increasingly sophisticated through the additions of cameras for recording procedures and pedals for adjusting magnification, all while offering high-quality images of the field [6]. However, operating microscopes still have room for improvement, as they can be bulky and inflexible. In contrast, loupes offer a lightweight and maneuverable alternative; however, they cannot change magnification or focal length [6]. Future surgical loupes might use cameras integrated into the frame, allowing for automated digital magnification of the surgical field. However, there would be significant engineering challenges for such development. Importantly, technology would have to make advanced assumptions to detect and focus objects of interest in the surgeon’s operative field. Although loupe-only “macro-level microsurgery” is possible with vessels above 1.5 mm in diameter [71], microscopes are still considered essential for many supermicrosurgery applications [71]. Extracorporeal telescopes, or exoscopes, employ a number of high-definition digital cameras around the operating room to provide surgeons with a magnified video display of the operative field [72]. This new technology offers an alternative to surgical loupes and the operative microscope. At MD Anderson Cancer Center in Houston, TX, USA, no significant differences in operative times and surgical complications were found between exoscopes and operative microscopes. However, surgeons reported less physical discomfort while using the exoscope, a factor that could make it a more attractive option to microsurgeons in the future [72].

### 5.2. Robot-Assisted Microsurgery

Robot-assisted procedures have appeared on the horizon of supermicrosurgery to help overcome the limitations of performing such procedures that challenge the natural boundaries of manual dexterity. In 2020, van Mulken et al. conducted a randomized trial to pilot robotic supermicrosurgery to treat breast-cancer-related lymphedema in 22 females [73]. This technology is not foreign to surgery; the da Vinci (Intuitive Surgical Inc., Sunnyvale, CA, USA) is used to perform minimally invasive procedures in a variety of subspecialities, mainly laparoscopic and thoracoscopic procedures [73]. However, in the field of microsurgery, the da Vinci poses some critical limitations, namely its poorer resolution at high levels of magnification [73]. These limitations led to the development of the MUSA (MicroSure, Eindhoven, The Netherlands), the world’s first robot dedicated for use in supermicrosurgery [73]. With the ability to dampen natural fine tremors, the MUSA can manipulate surgical instruments with ease and accuracy, as demonstrated in preclinical trials [74,75]. The study conducted in 2020 by van Mulken et al. was successful in showcasing the feasibility of the use of MUSA in accomplishing lymphovenous anastomosis, with comparable results at one and three months post-operatively to the manual procedure [73].

### 5.3. Sutureless Anastomotic Devices

Other avenues for improvement of vascular anastomosis could include forgoing sutured anastomosis entirely. For as long as surgeons have been sewing vessels together, innovators have tried to avoid sutures entirely [17]. Sutured anastomosis is time-consuming, and outcomes are highly dependent on the skill of the surgeon. There is a need to develop adhesives or devices that will remain in place permanently and securely without risk of thrombosis. Today, vascular shunts are utilized in the military to restore blood flow while wounded soldiers await definitive reconstruction outside of the combat theater [76,77]. However, these devices are only designed to remain in place temporarily. In 2016, Jose and colleagues [78] published preliminary laboratory results on a prototype anastomotic device made of a silk:glycerol bio-ink solution deposited in 40 µm monolayers. They showed that their device is potentially resorbable in vivo, potentially allowing for long-term placement, and could be secured in under a minute [78]. 

### 5.4. Tissue Adhesion

In 1982, Wintermantel described the creation of vascular anastomoses without sutures or permanent intralumenal implants [79]. He designed wire loops that would both approximate vessel ends and conduct an applied electrical current. The resulting heat would serve to fuse the vessels together. This technique showed remarkable success in Wintermantel’s rat model of carotid artery anastomosis, with 90% of anastomoses remaining patent after 30 days. The application of a muscle strip to these anastomoses with fibrin glue resulted in 100% patency after 6 months. The use of tissue adhesives alone has been a focus of ongoing research [80,81]. Adhesives such as cyanoacrylate are readily available in the clinical setting but, until recently, have not been successfully employed in vascular anastomosis, mainly due to challenges in maintaining luminal patency during their application. Recently, researchers have paired these adhesives with surgical stents made of poloxamers, water-soluble structures in current clinical use for drug delivery [82,83]. Poloxamers demonstrate thermo-reversibility between liquid and semisolid gel, allowing for them to temporarily hold vessels patent during application of tissue adhesives before returning to liquid state and allowing for restoration of blood flow. These stents have shown promise, resulting in increased patency and a wider lumen than conventional sutured anastomosis when paired with tissue adhesives [82,83,84]. Laser-assisted vessel-welding technologies, specifically those employing photothermal modalities, offer another potential alternative to traditional sutured anastomoses. These instruments produce heat by delivering light to endogenous chromophores on the vessel surface. This heat denatures and cross-links collagen molecules, allowing for adhesion without the use of tissue adhesives [85]. While this method has many potential advantages, such as minimizing foreign-body reaction and liquid-tight sealing, further research is required for this method to be brought into the operating room. The current modalities require stay sutures to obtain welding strengths equivalent to sutured anastomoses. Furthermore, this method poses the risk of damaging vessels through thermal diffusion [85].

## 6. Conclusions

**Figure 4:**
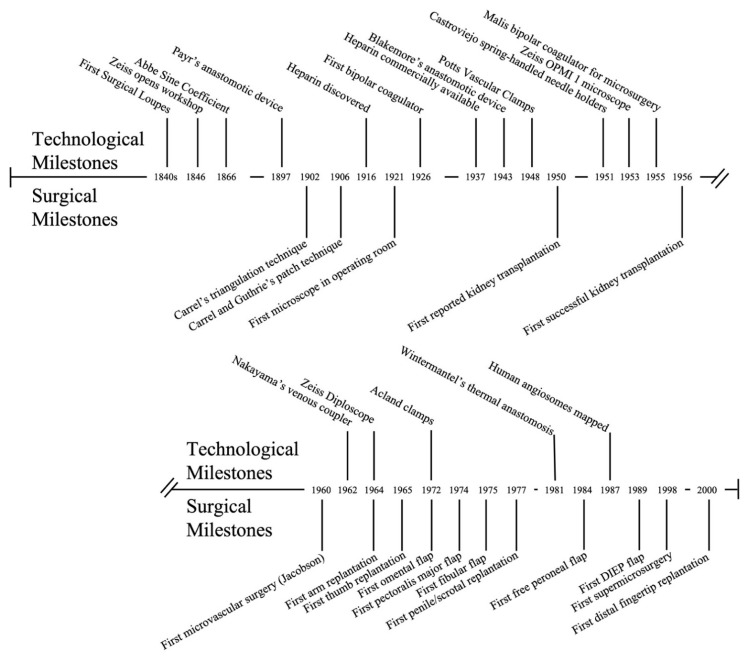
Technological and surgical milestones of blood vessel anastomosis.

Today, microvascular and vascular anastomosis can be performed on vessels of practically every size with consistently excellent results. We frequently remember the names of the surgeons who first demonstrated that these surgeries were possible. However, many of these successes were the culmination of decades of technological innovation (Figure 4). With the mass production of the laboratory microscope in the 1800s, Murphy, Carrel, and Guthrie were able to apply new understandings of vessel physiology to successfully achieve vessel anastomosis in the laboratory; by discovering heparin, Jay McLean allowed vascular anastomosis to be transitioned out of the laboratory and into the clinical setting; and after Carl Nylen brought a laboratory microscope into the operating room in the 1920s, Jacobson was able apply this technology to vessel anastomosis. As we look to push the boundaries of vascular anastomosis further, we can expect additional technological advances to improve our ability to place blood vessels of all sizes together with efficiency, patency, and improved clinical outcomes. 

## Figures and Tables

**Table 1:** Early innovations in microsurgery.

Year	Achievement	Innovator
1964	Zeiss Diploscope	Littman [6]
1964	First arm replantation	Malt and McKhann [50]
1965	First total thumb replantation	Komatsu and Tamai [45]
1965	Experimental thumb replantation	Buncke [51]
1966	First toe-to-thumb transplantation	Buncke [52]
1966	First total ear replantation	Buncke [53]
1972	Omentum transfer for scalp reconstruction	Buncke and McLean [54]
1973	First free skin flap	Daniel and Taylor [55]
1974	First pectoralis major transfer	Harii [56]
1975	First free fibular flap	Taylor [57]
1975	First free dorsalis pedis flap	McGraw [58]
1977	First penile and scrotal replantation	Tamai [59]
1982	First free scapular flap	Gilbert [60]
1984	First free peroneal flap	Yoshimura [61]

## Data Availability

Not applicable.
